# Optimization of the Therapeutic Approach to Patients with Sarcoma: Delphi Consensus

**DOI:** 10.1155/2019/4351308

**Published:** 2019-12-26

**Authors:** Rosa Álvarez Álvarez, Josefina Cruz Jurado, Xavier García del Muro Solans, Javier Lavernia Giner, Antonio López Pousa, Javier Martín-Broto, Claudia María Valverde

**Affiliations:** ^1^Hospital General Universitario Gregorio Marañón, Madrid, Spain; ^2^Hospital Universitario de Canarias, San Cristóbal de la Laguna, Tenerife, Spain; ^3^Instituto Catalán de Oncología (ICO), Hospitalet de Llobregat, Barcelona, Spain; ^4^Instituto Valenciano de Oncología (IVO), Valencia, Spain; ^5^Hospital de la Santa Creu i Sant Pau, Barcelona, Spain; ^6^Hospital Universitario Virgen del Rocío, Sevilla, Spain; ^7^Hospital Universitario Vall d'Hebrón, Barcelona, Spain

## Abstract

Soft tissue sarcomas (STS) constitute a heterogeneous group of rare solid tumors associated with significant morbidity and mortality. The evaluation and treatment of STS require a multidisciplinary team with extensive experience in the management of these types of tumors. National and international clinical practice guidelines for STS do not always provide answers to a great many situations that specialists have to contend with in their everyday practice. This consensus provides a series of specific recommendations based on available scientific evidence and the experience of a group of experts to assist in decision-making by all the specialists involved in the management of STS.

## 1. Introduction

Soft tissue sarcomas (STS) constitute a heterogeneous group of rare solid tumors that account for only 1-2% of all cancers in adults and 7% in children and adolescents [1]. The incidence of STS, some 80 histological subtypes of which have been identified to date [2], has risen over the past few years, with an average annual increase of 1.8% between 2002 and 2012 [3]. The relative mortality for patients with extremity and truncal STS at 5 and 10 years was determined to be 32.8% and 36.0%, respectively, and varied according to patient age, grade of the disease, stage at diagnosis, and comorbidities [4]. The selection of a treatment by a multidisciplinary team provides a basis for the management of the STS and is absolutely essential to the improvement of both the patient's prognosis and quality of life [5, 6]. Nevertheless, despite the advances made in our knowledge of the pathophysiology of the disease, the different national [7, 8] and international [2, 9] clinical practice guidelines, which have been developed using evidence collected from the literature, do not provide answers to a great many situations that specialists have to contend with in their everyday practice. In this context, the consensus of a group of experts can be a very useful tool. Therefore, this Delphi consensus on “Optimization of the therapeutic approach to patients with Sarcoma” provides a series of specific recommendations based on available scientific evidence and the experience of a group of experts to assist decision-making by all the specialists involved in the management of STS.

## 2. Methodology

The panel of experts of the Delphi consensus on “Optimization of the therapeutic approach to patients with Sarcoma” was made up by 20 specialists from all across Spain. They were chosen for inclusion on the panel due to their experience in the clinical management of patients with STS.

The Delphi method [10] was used to conduct the study in order to optimize the consultation process among the 20 panel members. To this intent, for questions measured on a metric scale, a level of agreement of more than 70% among all the experts consulted on the “top 4” (score of 7 or more points) was required to establish a consensus about each one of the questions asked and, conversely, a level of consensus equal to or greater than 70% was required for the “bottom 4” (score of 3 or less points) to determine a consensus about disagreement. On the other hand, for questions measured on a nominal scale, where respondents had to select one item or option from a nominal list containing more than two items, a level of consensus equal to or greater than 50%—“mode”—for the majority selected option was required in order to establish an agreement consensus. Lastly, for questions measured on an ordinal scale, where respondents were asked to rank the various items according to the degree of importance, a coefficient of variation equal to or less than 40% was required for an agreement consensus.

In total, there were 64 questions covering 7 different categories or specialty areas, which were drafted by the coordinators: localized high-risk disease, 10 questions (developed by J. M. B.); first-line treatment, 8 questions (developed by A. L. P.); second-line and subsequent therapy, 8 questions (developed by X. G. S.); metastasis surgery, 8 questions (developed by J. L. G.); retroperitoneal sarcomas, 10 questions (developed by R. A. A.); gynecological sarcomas, 10 questions (developed by J. C. J.); and disease follow-up, 10 questions (developed by C. V. M.).

The study methodology adopted a two-stage approach. During the first stage, which took place from September 27 to November 13, 2017, and which aimed at defining the level of consensus for the different issues that were raised, the 20 participating specialists (Appendix) responded anonymously to a totally structured online questionnaire that contained metric (majority), nominal, and ordinal questions. The members of the Scientific Committee, who were responsible for the systematic search of the literature for the drafting of the questions, did not respond to the questionnaire.

Questions that did not achieve a sufficient level of consensus were submitted for inclusion in the second stage, which took place online between December 19 and December 22, 2017, with the anonymous participation of 16 of the 20 specialists included in the initial sample. Again, the members of the Scientific Committee, who were responsible for the analysis and identification of the issues where the divergence of opinion was greatest, did not respond to the questions included in the second stage.

Finally, after the coordinators had put the resulting recommendations into writing, an in-person meeting was held on January 25, 2018, in which the experts voted to validate the recommendations.

## 3. Results and Discussion

### 3.1. Localized High-Risk STS

Localized high-risk (Table 1) STS are defined as those that meet the following criteria: G3, >5 cm, and deep-seated [11]. The panel of experts accepted this definition and, in turn, determined that since not all histological subtypes of G3 STS exhibit uniform behavior, cases in which G2 is established by needle biopsy (*Tru-Cut*) with necrosis of at least 50% seen on MRI should also be considered high risk. On the other hand, the grading system of the French Federation of Comprehensive Cancer Centers (FCCCs) [12] should not be used in cases of myxoid liposarcoma with transformation to a high-grade form with cellularity greater than 5%—spindle cell or round cell.

The results of the study by Tanaka et al. support the use of preoperative chemotherapy followed by postoperative chemotherapy consisting of three cycles of doxorubicin 60 mg/m^2^ and ifosfamide 10 g/m^2^ for localized high-risk extremity or trunk wall STS [13]. In this regard both, the noninferiority of three cycles of a full-dose conventional chemotherapy (epirubicin and ifosfamide) in comparison with five cycles and the association between response to therapy and better rates of overall survival (OS), particularly when Choi criteria are used for the evaluation, have been pinpointed in a randomized trial [11]. In light of the evidence, the experts recommend perioperative chemotherapy with anthracyclines and ifosfamide × 3 cycles for patients with localized high-risk extremity or trunk wall STS, with preference for neoadjuvant treatment because it seems to improve survival and adds potential prognostic information.

While neoadjuvant chemotherapy is not widely accepted in the approach to high-risk extremity and truncal STS, numerous studies support the use of radiation therapy in this situation, to the point where it is now considered a mainstay of treatment for high-risk patients. Still, the addition of chemotherapy to radiation therapy is associated with an improvement in the local response rate, reduction of the resection area, and a higher rate of preservation of the limb affected by STS [11, 14]. Moreover, and at the very least, potentially, chemotherapy can enhance the antitumor effects of radiation therapy [14]. For this reason, in an effort to improve the negative impact of potential R1 resection on patients with localized high-risk extremity and truncal STS, the panel of specialists recommends the use of preoperative chemoradiation therapy in this situation. In this case, in the event perioperative chemotherapy is used, the experts recommended the regimen of epirubicin 60 mg/m^2^/day on days 1-2 + ifosfamide 3 g/m^2^/day on days 1–3 every 21 days. When using this scheme, which can be delivered in an outpatient setting, monitoring of nadir counts is recommended at the very least in the first 2 cycles. This regimen has been evaluated in a joint study conducted by Sarcoma Research Groups in Italy, Spain, and France against histotype-tailored chemotherapy, and no advantage has been observed in the tailored regimen arm [15]. Additionally, MESNA (40% of the total daily ifosfamide dose at 0, 3, and 6 hours after the start of ifosfamide) and G-CSF support should be given in combination with this regimen. Alternatively, other equivalent regimens containing anthracycline and ifosfamide are considered adequate in this setting.

Likewise, the members of the panel agreed that patients with soft tissue masses >5 cm that have experienced recent growth or are deep-seated should be referred to a tertiary care facility or one known for its expertise in sarcomas where a needle biopsy (*Tru-Cut*) can be performed in order to establish the diagnosis and allow the case to be discussed with the multidisciplinary board.

After examining the evidence gathered from 33 articles, Kandel et al.'s review [16] concluded that patients with tumor-free margins have a better prognosis, so the authors determined that the goal of the surgery for extremity STS should be to achieve clear margins. Bearing this in mind, there was a high level of consensus among the panel experts when it came to recommending that surgical margins be prioritized over limb function in patients with high-risk extremity STS. If, in order to achieve adequate margins, limb function cannot be preserved, then radical surgery should be considered. Likewise, reoperation to widen margins should be carried out on patients with extremity STS who have positive margins after undergoing unplanned surgery.

Surgery is the treatment of choice for high-grade retroperitoneal sarcoma [17]. Also, the study by Hager et al. shows that adjuvant radiation therapy provides significant benefits in the five-year survival rates in this situation [18]. In this regard, the experts agreed that surgery alone should be recommended for the treatment of patients with high-grade retroperitoneal sarcoma and that the procedure should be carried out after proper planning at a tertiary care facility or one renowned for its expertise in sarcoma.

Finally, the factors influencing the recommendation of perioperative chemotherapy in patients with high-risk extremity or truncal STS are as follows: comorbidities, the presence of which has a very negative impact on the patient's prognosis [19]; histological subtype, since chemosensitivity varies very significantly according to the histology of the tumor [20, 21]; and age, as the effectiveness of chemotherapy with or without surgery and/or radiation therapy decreases progressively with age [22]. On this point, the consensus panel identified comorbidities, histological subtype, and age as factors to be evaluated prior to the administration of perioperative chemotherapy in high-risk STS and agreed it is not recommended for the treatment of patients over the age of 75.

### 3.2. First-Line Treatment

Anthracycline-based chemotherapy is the mainstay of first-line treatment (Table 2) for metastatic disease [2], specifically doxorubicin administered intravenously at a dose of 60–75 mg/m^2^ every 3 weeks [8, 23]. Likewise, the combination of doxorubicin and ifosfamide may be the treatment of choice for STS subtypes that are sensitive to ifosfamide and in those cases in which the patient has good functional status [2]. Judson et al. reported the results of a European Organization for Research and Treatment of Cancer (EORTC) randomized trial in first-line treatment (doxorubicin vs. doxorubicin + ifosfamide) and only recommend combination therapy when tumor shrinkage is needed, at the cost of increased toxicity (Lancet Oncology 2014, Judson) [24]. In this situation, in the phase II clinical trial conducted by Tap et al. to assess doxorubicin combined with olaratumab, a platelet-derived growth factor receptor alpha- (PDGFR*α*-) blocking antibody, the combination of olaratumab plus doxorubicin showed a significant improvement in OS compared with doxorubicin alone, although the response rate and progression-free survival were slightly lower [25]. Nevertheless, the results of a phase III trial (ANNOUNCE) presented at ASCO 2019 did not confirm that olaratumab + doxorubicin, followed by olaratumab monotherapy, improves OS over doxorubicin alone in patients with advanced STS.

Therefore, all of the panel experts agreed to recommend doxorubicin 75 mg/m^2^ administered as an intravenous infusion every 3 weeks as first-line treatment for metastatic disease. No consensus was reached for the combination of doxorubicin and ifosfamide in this situation.

The choice of first-line treatment for patients with advanced disease should be decided by a multidisciplinary team with extensive experience in the management of STS after taking patient age, functional status, comorbidities, and the tumor location and histological subtype into consideration [9]. Likewise, and in those cases where it is feasible, complete resection of the metastases is the treatment of choice, although the most definite experience has been with the excision of lung lesions [7]. Hence, the consensus panel determined that the most important factors to consider when selecting the best first-line treatment for a patient undergoing surgery for high-grade extremity STS with bilateral pulmonary metastases are as follows: histological subtype; comorbidities, age, and ECOG status of the patient; the potential for surgical treatment of the metastases; and progression-free survival greater than one year.

In the case of patients who underwent radical surgery for primary tumor in advanced extremity STS, and who have synchronous bilateral pulmonary metastases, the experts recommend administering doxorubicin alone or in combination with ifosfamide as first-line chemotherapy treatment of choice, in addition to surgical resection of the pulmonary metastases whenever possible. In this regard, the panel did not reach a consensus on the appropriateness of giving other types of chemotherapy (for example, docetaxel + gemcitabine), although there was a clear trend toward consensus disagreement. On the other hand, with respect to patients with resectable high-grade myxoid liposarcoma of the thigh with M1 lung disease, the experts reached a consensus with regard to starting standard chemotherapy using doxorubicin + ifosfamide.

In conclusion, if feasible, surgical excision of the lung metastases should be performed.

Some STS types could have a different sensitivity to chemotherapy or are considered chemoresistants, for example, alveolar soft part sarcoma (ASPS), a rare chemotherapy-resistant STS that most commonly occurs in adolescents and young adults; the consensus experts recommend that patients should always be enrolled in clinical trials or given first-line treatment with drugs that act against molecular targets, such as VEGFR inhibitors, given the low response to chemotherapy in this situation [26, 27]. Other chemoresistant histotypes were not included in this panel discussion.

Finally, the experts reached a consensus in identifying the administration of doxorubicin as the treatment of choice for elderly female patients diagnosed with uterine leiomyosarcoma with bilateral pulmonary metastases. In this case, it is recommended to use doxorubicin monotherapy and generally avoid local radiotherapy or chemotherapy in combination.

### 3.3. Second-Line Treatment and Subsequent Therapy

Second-line treatment (Table 3) for advanced STS is always palliative, so it should not be administered to asymptomatic patients [8]. Although symptomatic patients should be considered preferentially for inclusion in clinical trials, different palliative options exist to control symptoms, such as local radiation therapy, supportive medical treatment, and supportive chemotherapy [8]. In this context, after progression to first-line chemotherapy, the members of the panel recommend initiating treatment with the most active therapy available for each case, as in subsequent lines efficacy will decrease, and to prefer the less toxic alternatives as they have a less negative impact on the quality of life. The experts also determined that optimal treatment should include the maximum possible number of drugs or regimens, to be used sequentially following failure of a previous treatment, provided the patient maintains adequate functional status.

The selection of second-line treatment for STS should be decided on an individual basis, taking into account both the characteristics of the patient and the histological subtype of the sarcoma, tumor burden, and expected toxicity [28, 29]. Along these lines, the consensus panel experts identified comorbidity, age, and functional status of the patient as the main factors to be assessed when choosing second-line chemotherapy. Other aspects to consider, in decreasing order of importance, are histological subtype, toxicity to first-line chemotherapy, and the response to the first-line chemotherapy.

*Trabectedin* has consistent activity in the different types of STS, so that it can be considered for administration after failure of first-line therapy with anthracyclines or in patients for whom anthracycline-based regimens are contraindicated [28]. Moreover, once the need for a change in treatment has been confirmed, trabectedin has a relatively favorable toxicity profile compared to other chemotherapy agents [28]. Hence, the members of the consensus panel agreed with the guidelines [2, 8] by considering that trabectedin should always constitute an option when assessing second-line treatment for the STS. On the other hand, in addition to showing numerous data on its activity in sarcoma subtypes other than leiomyosarcoma or liposarcoma, particularly in the case of metastatic synovial sarcoma [30], the studies have also confirmed the effectiveness of trabectedin in modulating the transcription of oncogenic fusion proteins, so it may be particularly useful in the treatment of sarcomas derived from specific translocations such as myxoid liposarcoma and Ewing sarcoma [28]. The presence of *t*(12;16) or *t*(12;22) translocations leads to FUS-CHOP or EWS-CHOP fusion proteins in myxoid liposarcomas [31]. Consequently, in addition to underline its use for the palliative treatment of synovial sarcoma, the consensus experts agreed that treatment with trabectedin is an especially valid option for myxoid liposarcoma. Likewise, the panel also considers that the reintroduction of trabectedin after progression in patients who previously achieved a good response may be an option to consider for selected patients. It is also recommended to maintain treatment until disease progression in patients with clinical benefit and acceptable tolerability.

Trabectedin discontinuation after the sixth treatment cycle in patients with advanced soft tissue sarcoma who are free from disease progression significantly shortens progression-free survival compared with patients who receive trabectedin continuously. On the basis of these results, it is not recommended a drug holiday in patients who benefit from trabectedin in terms of tumor control after six courses of treatment [32].

The response rate for ifosfamide as monotherapy for second-line or subsequent treatment of STS varies between 4.8% and 62.5% across series [33], with an extremely poor rate for leiomyosarcomas in particular [28]. Similarly, the studies have shown that, in metastatic STS, the response rate with ifosfamide is around 20–25% and mean OS is close to 12 months, which is comparable with outcomes for doxorubicin [34]. Conversely, a retrospective analysis conducted by the EORTC STS Group found that response rates were higher for ifosfamide-based regimens versus doxorubicin-based regimens in the second-line treatment of synovial sarcomas [35]. In view of the above, and given the toxicity and complex administration schedule of ifosfamide [28], the panel recommended treatment with ifosfamide be considered only for certain subtypes and for those cases in which the therapeutic objective is to achieve a rapid response, while other more appropriate alternatives should be considered for patients with leiomyosarcomas.

Pazopanib is the only oral agent approved for the treatment of high-grade STS and can be considered an acceptable option for different STS subtypes, excepting liposarcomas, where the response rate is extremely poor [29], after failure of first-line therapy with anthracyclines [28]. Indeed, consensus experts agreed in calling attention to its toxicity profile, which differs from that associated with chemotherapy agents and may prove to be an advantage in patients with significant toxicity to previous lines, although it should never be considered for administration as second-line therapy for liposarcoma and requires paying special attention to the risks of hepatotoxicity, high blood pressure (HBP), and hyperthyroidism. Unsurprisingly, the standard oral dose of 800 mg of pazopanib is associated with side effects common to many anti-VEGF therapies, such as HBP, thrombosis, proteinuria, hypothyroidism, cardiac dysfunction as well as gastrointestinal and hepatic disorders [36], especially the elevations in transaminase levels [37]. The PALETTE study, a randomized, double-blind, placebo-controlled phase III study conducted by the EORTC STS Group in patients with metastatic STS, found an incidence of 7% of HBP in the control group and 41% in the pazopanib arm [38]. On the other hand, the article by In et al. showed that pazopanib has activity as second- and third-line treatment for synovial sarcomas, undifferentiated pleomorphic sarcomas, and malignant peripheral nerve sheath tumors and may even be considered for administration as second-, third-, and even fourth-line therapy for select patients with leiomyosarcoma [29]. In this regard, the members of the panel considered that pazopanib constitutes an adequate therapy option for third- or fourth-line treatment of nonadipocytic STS.

On another note, the authors did not reach a consensus when considering second-line treatment with eribulin as an effective therapy option for leiomyosarcoma. In that respect, the improvement in OS with eribulin as compared with dacarbazine was observed only in patients with liposarcoma (OS 15.6 vs. 8.4 months, HR: 0.511, 95% CI: 0.3446–0.754), but not in those with leiomyosarcoma (OS 12.7 vs. 13 months, HR: 0,927; 95% CI: 0.714–1.203) [39]. Moreover, the benefit of treatment with eribulin for STS was limited to overall survival, with no improvement found for progression-free survival [40]. However, the experts were unable to reach a consensus when determining whether the fact of not being associated with an increase in progression-free survival is a drawback for its use in the clinical setting.

Regarding combination therapy for second-line treatment in advanced STS, there are a few data on clinical trials comparing different schedules. The combination of gemcitabine and dacarbazine has activity in patients with previously treated STS [41–43], with a better tolerance profile than the one observed with the combination of gemcitabine and docetaxel in the SARC002 study [44]. To be specific, the rate of progression-free survival (PFS) after three months of treatment with the combination of gemcitabine and dacarbazine was 46% in the study by Losa et al. [41] and 56% in the one conducted by García del Muro et al. [42]. Accordingly, the experts noted that this combination, besides having a better tolerance profile, in general than the combination of gemcitabine and docetaxel, may constitute a treatment alternative to be considered not only for leiomyosarcoma, but also for the rest of STS. In this regard, the phase II clinical trial conducted by García del Muro et al., which compared gemcitabine plus dacarbazine versus dacarbazine alone, found higher and statistically significant OS rates (16.8 months vs. 8.2 months) and progression-free survival rates for the combination therapy [42]. As a result, and taking its lower activity in comparison with other available alternatives into account, the panel's members concluded that dacarbazine should no longer be used for control arm patients in randomized second-line clinical trials.

### 3.4. Metastasis Surgery

Although the existing international consensus is that patients with STS should be referred to a specialist sarcoma center for treatment, in our country 50% of STS larger than 5 cm are operated on without a previous diagnosis of sarcoma being established [8]. In this regard, all of the members of the panel agreed with clinical practice guidelines [5, 45] requiring that patients with advanced STS be managed at centers with a sarcoma specialist multidisciplinary team—including radiologists, pathologists, medical and radiation oncologists, surgeons, and psychologists—with experience in the management of these types of tumors. The experts also reached a consensus on the need to propose surgical removal of the primary tumor to all patients with good functional status who are diagnosed with resectable STS and have multiple asymptomatic pulmonary metastases (Table 4).

The objective of any treatment should be to improve the quality of life for the patient. In this sense, palliative surgery can provide significant relief of symptoms, especially of pain, in properly selected patients with metastatic STS [46]. For this reason, the members of the consensus panel considered palliative surgery to be a good option for the treatment of patients with advanced STS who are progressing on systemic treatment and who have symptomatic metastases, regardless of their location, recommending palliative surgery for metastases as prophylaxis for symptoms or complications—among others, fractures, gastrointestinal compressions, and hemorrhages—as long as it can be effective for their palliation.

Although to date no phase III clinical trials have been conducted to compare surgery to the other therapy options in local treatment of metastatic STS, numerous retrospective studies suggest that radical tumor resection provides increased survival in this situation [47]. Radiation therapy and thermal radiofrequency ablation, as shown by Stragliotto et al. and Lindsay et al., represent valid alternatives for the treatment of metastasis when surgery is not indicated or feasible.

In light of this evidence, experts identified SBRT and surgery as the most reliable local treatment options for oligometastatic disease, although no consensus was reached with regard to thermal ablation.

Patients with metastatic STS generally have an extremely poor prognosis despite whatever treatment they are receiving. Patients with isolated lymph node metastases should be considered as potentially curable and should undergo radical surgery for both the primary tumor and the metastases. This approach would be not applicable for those patients with pulmonary metastases, in which the treatment objective is less clear since the probability of cure is much more remote. Therefore, in case of pulmonary metastases, the possibility of including the patient in a clinical trial or initiating chemotherapy with palliative intent should be evaluated [46]. In view of the above, the members of the consensus panel recommended including all patients with bilateral pulmonary metastases from STS in whom complete resection cannot be achieved in clinical trials. Finally, a meta-analysis by Treasure et al. found a 5-year survival rate of 25% after a first pulmonary metastasectomy for patients with STS, with better survival found in cases where there were fewer metastases and longer intervals between diagnosis of STS and the appearance of metastases [50]. That notwithstanding, the authors underscore that there is no evidence to support that metastasectomy will lead to an increase in survival for STS patients, suggesting that the higher survival observed for patients undergoing surgery vs. those who had no surgery can be explained by the fact that the patients selected for this type of procedure are usually those who have a better prognosis, rather than due to the effect of metastasectomy in itself. Keeping this in mind, the panel's experts recommended considering pulmonary metastasectomy only for cases in which the pulmonary metastases, whether unilateral or bilateral, are resectable, and always taking into account patient functional status and the progression-free interval.

### 3.5. Retroperitoneal Sarcoma

The guidelines by the Grupo Español de Investigación en Sarcomas (GEIS) (*Spanish Sarcoma Research Group*) [8] make it mandatory to perform a core needle biopsy for all retroperitoneal sarcomas (Table 5) if neoadjuvant therapy is planned, other abdominal tumors are evaluated for differential diagnosis, or there is reasonable clinical suspicion of sarcoma in the presence of a retroperitoneal lesion. In addition, and as occurs for most STS, fine-needle aspiration (FNA) cytology is not suitable for a primary diagnosis of retroperitoneal sarcomas. In the same line, the guidelines published in 2018 by ESMO and the European Reference Network for Adult Rare Solid Cancers (EURACAN) [2] acknowledge the need for biopsy prior to starting any treatment in order to establish a pathological diagnosis of retroperitoneal sarcoma and thus facilitate making both present and future treatment decisions. The standard procedure consists in using a coaxial needle with an appropriate gauge (14–16 G) to obtain a multiple core biopsy, a procedure associated with minimal risk of dissemination in the needle tract. Accordingly, the panel of experts recommended performing core needle biopsy for retroperitoneal sarcomas whenever neoadjuvant radiation and/or chemotherapy is planned and determined that the risk of dissemination in the needle tract, which is minimal, should not be a reason for not performing this procedure and that under no circumstances should FNA be performed instead, as the latter provides little diagnostic information and can only cause delays in starting potential therapy.

Because of their complexity, retroperitoneal sarcomas should always be treated in centers that have multidisciplinary teams with experience in the management of these types of tumors [51]. Surgery is the mainstay of treatment for primary lesions and the only curative option for retroperitoneal sarcoma, and it must be performed by a surgeon with specific experience in this type of STS [2]. Indeed, the surgical team must be prepared, or have the necessary help, to perform techniques such as bowel resection, nephrectomies, or diaphragm reconstruction [8]. For these reasons, the members of the panel determined that patients with retroperitoneal sarcoma must always be referred to a tertiary care facility or one known to have a multidisciplinary team/committee with extensive surgical experience in the management of tumors at this location.

In this context, after a patient has been referred for assessment following inadequate primary surgery—no en-bloc resection, R0, and so on—of retroperitoneal sarcoma at a hospital center lacking experience, the treatment approach will depend on the grade of the disease. Hence, in cases of low-grade retroperitoneal sarcoma, the authors recommend performing a CT scan of the thorax, abdomen, and pelvis (CT TAP scan) and set up a schedule to follow up closely in the absence of clear disease data until development of the macroscopic lesions, at which time a new surgery should be planned. On the other hand, in cases of high-grade disease, treatment should be tailored according to patient characteristics. Likewise, with regard to the treatment of local recurrence of retroperitoneal sarcoma, rescue surgery should only be considered for cases in which the disease is resectable and R0 can be achieved.

The level of evidence is not enough to support the routine use of radiation therapy complementary to surgery. Although retrospective studies suggested that adding radiotherapy to treatment could reduce the risk of local recurrence, there are no randomized studies confirming it. The randomized, phase III STRASS (EORTC-STBSG 62092–22092) failed to demonstrate the benefit of preoperative radiotherapy in the treatment of localized retroperitoneal sarcomas. However, in the exploratory analysis, a possible benefit of this treatment was described in the LPS subgroup [52]. The experts on this consensus panel agreed to the determination that when a decision is made to use complementary radiation therapy for patients who have potentially resectable retroperitoneal sarcoma, preoperative radiation therapy should always be administered as long as it is possible to meet with the radiation field requirements.

Although to date no randomized clinical trials to compare neoadjuvant therapy with radical surgery alone for retroperitoneal sarcoma have been conducted, neoadjuvant treatments such as chemotherapy or external beam radiation therapy (EBRT) are safe for select patients, so their use can be assessed subject to a review by the multidisciplinary team [2]. The GEIS guidelines establish that even though, generally speaking, neoadjuvant chemotherapy is not recommended, it may be indicated in specific cases when there are realistic expectations of improving resectability [8]. On this point, the panel members agreed that neoadjuvant chemotherapy should be considered for high-grade retroperitoneal sarcomas with potential sensitivity to chemotherapy (leiomyosarcoma, angiosarcoma, synovial sarcoma, etc.) that are at the limits of resectability.

Given the absence of evidence to show increase in survival, adjuvant chemotherapy should not be administered on a routine basis for surgically resected retroperitoneal sarcomas. The study by Datta et al. [53] compared OS associated with surgery alone vs. adjuvant chemotherapy in 767 patients with retroperitoneal sarcomas who had been surgically resected. The results showed that the use of adjuvant chemotherapy was associated with decreased long-term survival (median OS: 47.8 vs. 68.9 months, *p*=0.017; HR: 1.30, 95% CI: 1.05–1.61). Hence, the experts determined that since adjuvant chemotherapy cannot be considered standard treatment for resected retroperitoneal sarcomas, in the event that it is considered as an option in some individual cases, the factors that should be assessed are, in decreasing order of importance, risk of relapse, histology, and histological grade. Furthermore, should such a treatment choice be made, a regimen consisting of anthracycline-ifosfamide would be administered.

### 3.6. Gynecological Sarcomas

When surgical resection is not feasible, the treatment for advanced recurrent endometrial stromal sarcoma (ESS) is administration of systemic hormone therapy with palliative intent [54]. According to the established definition, ESS is a low-grade sarcoma characterized by a high expression of estrogen (ER, 40–100%) and progesterone (PgR; 60–100%) receptors [55], which is why hormone treatments to reduce estrogen levels play a central role in the management of ESS and are more effective than the different chemotherapy regimens [56]. In this regard, given their efficacy and tolerability, both the aromatase inhibitor letrozole as well as progestins could be the treatment of choice for patients with recurrent or residual unresectable ESS [57]. Conversely, in cases in which the disease has become resistant to estrogen deprivation, chemotherapy should be indicated, although with less favorable outcomes [55]. On the basis of this evidence, the experts determined that, for the treatment with palliative intent of unresectable ESS that expresses estrogen receptors, the first therapy option should be hormone treatment using aromatase inhibitors rather than anthracycline-based chemotherapy (Table 6).

The mainstay of treatment for localized ESS is radical abdominal hysterectomy with bilateral salpingo-oophorectomy [56]. Given the elevated expression of ER and PgR receptors, hormone replacement therapy after surgery is contraindicated [58], as is adjuvant radiation therapy, whose use in this situation has been shown to be ineffective [59]. Very few studies have been conducted to evaluate the effectiveness of chemotherapy in this situation. The work by Kim et al. found that adjuvant chemotherapy had no effect on the prognosis of patients with stage I low-grade ESS [60]. Likewise, the multivariate analysis by Feng et al. showed that use of multiple chemotherapy regimens may improve progression-free survival in low-grade localized ESS, although the results were inconclusive [61]. Similarly, a lack of clinical trials means that, at present, we cannot determine whether hormone therapy can be beneficial in the general approach to all low-grade ESS or whether it should only be administered to patients at high risk for recurrence [61]. In view of the situation, the US National Comprehensive Cancer Network (NCCN) recommends close observation without treatment after surgery for stage I ESS [62]. On their side, the consensus panel experts determined that the administration of hormone therapy with letrozole should not be recommended after radical surgery for localized low-grade ESS expressing estrogen receptor.

Early and complete resection constitutes the best treatment option for uterine leiomyosarcoma confined to the neck and body of the uterus [63, 64]. The largest case series study performed to date shows that the ability to achieve complete tumor cytoreduction is associated with a statistically significant increase in disease-free survival [65]. Under these circumstances, the current clinical practice is to perform hysterectomy with bilateral oophorectomy. However, the incidence of occult ovarian (<4%) and lymph node (<3%) metastases in uterine leiomyosarcoma is very low and they are usually associated with extrauterine disease [66]. In this respect, the study by Kapp et al. failed to show a significant difference in 5-year disease-specific survival between patients who underwent or did not undergo bilateral oophorectomy at the time of hysterectomy [67]. In consequence, the panel of experts recommended performance of simple hysterectomy as the surgical treatment of choice for uterine leiomyosarcoma and stated that bilateral oophorectomy is not necessary. On the other hand, in case of advanced, unresectable uterine leiomyosarcoma, the decision to initiate palliative systemic treatment will be determined by the grade of disease.

The clinical practice guidelines published by the British Sarcoma Group determine that any patient with a suspected STS should be referred to a diagnostic center for assessment by a specialist sarcoma multidisciplinary team expert in the management of these types of tumors [45]. Likewise, the joint guidelines published by the European Sarcoma Network Working Group (ESNWG) and the European Society for Medical Oncology (ESMO) clearly state that a multidisciplinary approach is mandatory for all patients with STS and must involve pathologists, radiologists, surgeons, radiation therapists, and oncologists, among other specialists, and that management should be carried out at a tertiary care facility or a center known for its expertise in sarcomas and having a multidisciplinary team/committee and treating a high number of patients every year [5]. Thus, the consensus panel recommended that all gynecological sarcomas be referred to an interdisciplinary team for assessment, to thus reach agreement on what type of treatment would be best for the individual patient, determining the need to set up some form of consultation during the decision-making process before the gynecologists, who are usually responsible for the diagnosis of these tumors, make the surgical decision.

### 3.7. Disease Follow-Up

After the treatment of the primary STS, 11–14% of patients develop local recurrences and between 18% and 50% end up developing metastases [45, 68]. The 2018 guidelines published by the US National Comprehensive Cancer Network (NCCN) [9] recommends local follow-up of patients with high-grade STS every 3–6 months during the first 2–3 years, every 6 months until the fifth year, and annually from the sixth year onwards. For patients with low-grade STS, local follow-up is recommended every 3–6 months during the first 2–5 years and annually from the sixth year onwards. For its part, the ESMO-EURACAN guidelines [2] recommend that surgically treated patients with intermediate-high-grade STS be followed every 3-4 months during the first 2-3 years, every 6 months until the fifth year, and annually from the sixth year onwards. However, both the NCCN and the ESMO and the EURACAN acknowledge that there is a lack of evidence in the literature regarding the effectiveness of these recommendations, indicating the need for prospective clinical trials to be conducted in this regard (Table 7).

A study by Sawamura et al. [69] retrospectively reviewed the records of 867 patients with STS who were treated surgically, with the aim of evaluating the time elapsed between resection of the tumor and the diagnosis of local recurrences, the time elapsed between surgery and the diagnosis of distant metastases, and the difference in those parameters based on tumor size and grade. Low-grade STS consistently recurred during follow-up and developed metastasis very rarely, so the authors recommended follow-up—physical examination and MRI—every 6 months during the first 5 years and annually from the sixth year until the tenth year. On the other hand, high-grade STS had a higher rate of local recurrence and metastases than low-grade STS, especially during the first two years, leading the authors to recommend follow-up—physical examination and MRI—every 3 months during the first two years, then every 6 months up to the fifth year, and annually from the sixth year until the tenth year. Regardless of the grade, 95% of local recurrences and metastases were detected during the first 9 years of follow-up, so the authors do not see any justification for continuing the follow-up after the tenth year.

The recommendations of the panel of experts regarding the follow-up of the disease are included in Table 6.

There is a paucity of evidence in the literature regarding the effectiveness of different follow-up strategies, including the use of MRI or CT scans. Indeed, to date, no studies have been published to show that the use of CT scans during routine follow-up of patients with STS can be associated with an improvement in prognosis [9]. The ESMO-EURACAN guidelines note that although the use of MRI to detect local recurrences and CT scans for pulmonary metastases may allow these episodes to be found earlier, it has not been shown that this is beneficial or cost-effective, compared with the clinical assessment of the primary tumor and regular chest X-rays [2]. Keeping this in mind, the members of the panel recommend performing CT scans every 12 weeks in the follow-up of patients with metastatic disease receiving active treatment or no treatment, but whose general condition is good and lung CT scans every 3 months during the first two years and every 6 months after the third year after resection of the pulmonary metastases.

Likewise, consensus experts identified the appearance or worsening of symptoms as the primary factor for considering that a patient has progressed on a treatment and needs to change to a different one, followed by progression based on RECIST criteria.

Finally, several studies have confirmed the usefulness of PET-CT scans to predict the course of the disease and monitor response to therapy [70, 71], although their use has to be seen as standard for the majority of patients. The ESMO-EURACAN guidelines note that it is mandatory to perform an abdominal CT scan and a bone scan or an ^18^F-FDG PET-CT scan in order to confirm that the lung metastases are “isolated” [2]. In this regard, the expert consensus identified PET-CT scans as an especially useful tool prior to the resection of pulmonary metastases from STS.

## Figures and Tables

**Table 1:** Treatment recommendations for localized high-risk soft tissue sarcomas.

Recommendation	Phase	Type of consensus (% agreement)
Tumor must be G3, >5 cm, and deep-seated for classification as high-risk extremity or truncal STS	1	Yes (mode: 70%)
Treatment for high-risk extremity or truncal STS consists in the administration of the following:		
(i) 3 cycles of perioperative chemotherapy with full-dose epirubicin + ifosfamide	1	Yes (mode: 75%)
(ii) Preferably in the neoadjuvant setting	1	Yes (mode: 60%)
Use of preoperative (vs. postoperative) radiation therapy is recommended for high-risk extremity or truncal STS, provided that the possibility of resection is marginal (increased risk of R1)	1	Yes (mode: 70%)
The recommended perioperative chemotherapy regimen for high-risk extremity or truncal STS is as follows: epirubicin 60 mg/m^2^/day as a 20 min infusion on days 1-2 and ifosfamide 3 g/m^2^/day as a 3-hour infusion on days 1–3 or equivalent regimens	1	Yes (mode: 100%)
Patients with a soft tissue mass >5 cm that have experienced recent growth or are deep-seated should be referred to a tertiary care facility or one renowned for its expertise	1	Yes (mode: 90%)
Adequate surgical margins should be prioritized over limb function in patients with high-risk extremity or truncal STS	1	Yes (mode: 90%)
If positive margins are confirmed, reoperation to widen surgical margins should always be attempted in patients with high-risk extremity or truncal STS	1	Yes (mode: 85%)
Standard treatment for retroperitoneal sarcoma is surgery carried out after proper planning at a tertiary care facility or one renowned for its expertise	1	Yes (mode: 50%)
Before administering perioperative chemotherapy to patients with high-risk extremity or truncal STS, the following risk factors must be considered:		
(i) Comorbidities	2	Yes (93.8)
(ii) Histological subtype	2	Yes (81.3)
(iii) Age	2	Yes (68.8)

**Table 2:** Recommendations for first-line treatment.

Recommendation	Phase	Type of consensus (% agreement)
The first-line treatment of choice for metastatic disease is doxorubicin 75 mg/m^2^ administered as an IV infusion every 3 weeks	1	Yes (100)
The most important factors to consider when selecting the best first-line treatment for patients undergoing surgery for high-grade extremity STS with bilateral pulmonary metastases are as follows:		
(i) Histological subtype	1	Yes (90)
(ii) Comorbidities, age, and ECOG performance status of the patient	1	Yes (90)
(iii) Potential for surgical treatment of the metastases	1	Yes (90)
(iv) Duration of progression-free interval	1	Yes (80)
The first-line treatment of choice for patients undergoing surgery for high-grade extremity STS with bilateral pulmonary metastases is as follows:		
(i) Doxorubicin alone	1	Yes (90)
(ii) Doxorubicin + ifosfamide	1	Yes (75)
(iii) Surgical resection of the pulmonary metastases	1	Yes (75)
The treatment of choice for resectable high-grade myxoid liposarcoma of the thigh with M1 lung disease is chemotherapy with doxorubicin + ifosfamide	2	Yes (68.8)
Standard chemotherapy regimens are not recommended for patients with metastatic alveolar soft tissue sarcoma, given their limited activity; drugs that act against molecular targets, such as VEGFR inhibitors, should be used instead	1	Yes (75)
Enrollment in a clinical trial should always be considered for these patients	1	Yes (85)
The first-line treatment of choice for elderly female patients with metastatic uterine leiomyosarcoma should be the following:		
(i) Doxorubicin	2	Yes (68.7)
(ii) Doxorubicin + ifosfamide	2	No (87.5)
(iii) Local radiation therapy	2	No (68.8)

**Table 3:** Recommendations for second-line and subsequent therapy.

Recommendation	Phase	Type of consensus (% agreement)
Optimal treatment following progression to first-line chemotherapy should		
(i) Include the maximum possible number of drugs or regimens, to be used sequentially following failure of a previous treatment, provided the patient maintains an adequate performance status	1	Yes (85)
(ii) Be initiated with the most active therapy available for each case, taking into account both histotype and specific patient characteristics, as in subsequent lines efficacy will decrease	1	Yes (90)
(iii) Given the palliative context, give priority to less toxic alternatives as they have a less negative impact on the quality of life	1	Yes (80)
The following factors should be considered when selecting second-line treatment (in decreasing order of importance):		
(i) Comorbidity, age, and performance status of the patient	2	Yes (mean: 3.8; CV: 11.9%)
(ii) The sarcoma histological subtype	2	Yes (mean: 3.6; CV: 25.2%)
(iii) Toxicity to first-line chemotherapy	2	Yes (mean: 1.6; CV: 38.1%)
(iv) Response to first-line chemotherapy	2	Yes (mean: 1.6; CV: 40.3%)
(1) Trabectedin has a favorable toxicity profile compared to classic chemotherapy agents and constitutes a second-line option that is always worth considering when treating STS	1	Yes (90)
(2) Numerous data on activity in sarcoma subtypes other than leiomyosarcoma or liposarcoma, such as synovial sarcoma, support the possibility of also using trabectedin to treat these STS subtypes	1	Yes (75)
(3) Treatment with trabectedin is an especially recommended option for myxoid liposarcoma	1	Yes (100)
(4) Treatment with trabectedin until progression may extend time to progression and is an option worth considering for patients as it shows clinical benefit and acceptable tolerance	1	Yes (95)
(5) Rechallenge with trabectedin after progression in patients who previously achieved a good response may be an option to consider for select patients	1	Yes (75)
(6) Given its toxicity and complex administration schedule, ifosfamide is preferably indicated for certain subtypes and for cases in which the therapeutic objective is to achieve a rapid response	2	Yes (68.8)
(7) Other more appropriate alternatives than ifosfamide should be considered for second-line treatment of leiomyosarcoma	2	Yes (93.8)
(8) Due to its toxicity profile, which is different from that of chemotherapy agents, pazopanib may prove to be an advantage in patients with significant toxicity to previous lines	1	Yes (90)
(9) Pazopanib should never be used for the treatment of liposarcomas	2	Yes (87.5)
(10) Pazopanib constitutes an adequate option for second-line and subsequent treatment of nonadipocytic STS	2	Yes (68.8)
(11) Blood pressure and hepatic and thyroid function should be monitored during treatment with pazopanib	2	Yes (93.8)
(12) Overall, the combination of gemcitabine and DTIC has a better tolerance profile than gemcitabine plus docetaxel	1	Yes (95)
(13) The combination of gemcitabine and DTIC may be a treatment alternative to be considered for leiomyosarcoma as well as the rest of STS	1	Yes (90)
(14) Given its lower activity compared to other available drugs, DTIC should no longer be used for standard control arm patients in randomized second-line clinical trials	1	Yes (70)

**Table 4:** Recommendations for metastasis surgery.

Recommendation	Phase	Type of consensus (% agreement)
Patients with advanced sarcoma should be managed at centers with a multidisciplinary team (radiologists, pathologists, surgeons/traumatologists, medical and radiation oncologists, psychologists, and physiotherapists) specializing in sarcoma treatment	1	Yes (100)
Primary tumor surgery should always be considered for patients with ECOG ≤2 who have resectable soft tissue sarcoma and multiple asymptomatic pulmonary metastases	1	Yes (mode: 50%)
Palliative surgery for metastases may be an option for patients with advanced sarcoma who are progressing on systemic treatment and who have symptomatic metastases	2	Yes (68.8)
The most reliable local treatment options for oligometastatic disease are as follows:		
(i) Surgery	1	Yes (80)
(ii) Stereotactic body radiation therapy (SBRT)	2	Yes (87.5)
For the purpose of preventing symptoms or complications, palliative surgery for metastases from sarcoma should always be performed as long as it will be effective for palliation	2	Yes (mode: 85%)
Inclusion in a clinical trial should be considered for all patients with pulmonary metastases from STS when palliative treatment may be an option and it proves impossible to achieve an R0 resection	1	Yes (95)
Pulmonary metastasectomy should only be considered when the pulmonary metastases, whether unilateral or bilateral, are resectable, and always taking into account the following factors:	1	Yes (mode: 75%)
(i) ECOG	2	Yes (mode: 50%)
(ii) Progression-free interval	2	Yes (mode: 50%)

**Table 5:** Recommendations for retroperitoneal sarcomas.

Recommendation	Phase	Type of consensus (% agreement)
Performance of core needle biopsy for the diagnosis of retroperitoneal sarcoma (RS)		
(i) Must always be carried out if neoadjuvant therapy is planned (radiation therapy and/or chemotherapy)	1	Yes (95)
(ii) It must not be replaced by FNA, as it provides little diagnostic information and can cause delays in starting potential treatment	1	Yes (90)
(iii) Has minimal risk of dissemination in the needle tract, so this should not be a reason for not performing this procedure	1	Yes (75)
Patients with RS should always be referred to a tertiary care facility or one renowned for its expertise and known to have a multidisciplinary team/committee with extensive surgical experience in the management of tumors at this location	1	Yes (mode: 85)
The procedure to be followed in patients who are referred for assessment after inadequate RS surgery (no en-bloc resection, R0, etc.) performed at a hospital center lacking the necessary experience depends on the grade:		
(i) Low-grade RS: performance of CT TAP scan and follow up closely in the absence of clear disease data until development of macroscopic lesions, at which time a new surgery should be performed	1	Yes (mode: 50%)
(ii) High-grade RS: treatment should be tailored to each case, as there are no clear recommendations in this regard	1	Yes (mode: 50%)
Rescue surgery should be considered as treatment for local RS recurrence in cases in which the disease is resectable and R0 can be achieved	1	Yes (mode: 80%)
Complementary radiation therapy cannot be used as standard treatment for patients who have potentially resectable RS; if after individualized assessment it is indicated, preoperative radiation therapy will be administered in every case as long as it is possible to meet the radiation field requirements	2	Yes (93.8)
Neoadjuvant chemotherapy should be considered for high-grade RS with potential sensitivity to chemotherapy (leiomyosarcoma, angiosarcoma, synovial sarcoma, etc.) that are at the limits of resectability	1	Yes (75)
Given the absence of evidence to show increase in survival, adjuvant chemotherapy cannot be considered standard treatment for resected RS; hence, if considered as an option in individual cases, the factors that should be assessed are (in decreasing order of importance) as follows:		
(i) Risk of relapse	2	Yes (mean: 4.0; CV: 28.9%)
(ii) Histology	2	Yes (mean: 3.9; CV: 22.8%)
(iii) Histological grade	2	Yes (mean: 3.3; CV: 32.5%)
In the event that the decision is made to treat resected RS with adjuvant chemotherapy, the regimen to be administered would consist of a combination of an anthracycline and ifosfamide	1	Yes (mode: 50%)

**Table 6:** Recommendations for gynecological sarcomas.

Recommendation	Phase	Type of consensus (% agreement)
First-line palliative treatment for unresectable stromal sarcoma should be hormone treatment with letrozole	1	Yes (70)
After surgery for localized stromal sarcoma, hormone treatment with letrozole should be administered	2	No (75)
Simple hysterectomy is the surgical treatment of choice for uterine leiomyosarcoma	2	Yes (93.8)
The histological grade of the uterine leiomyosarcomas determines the type of palliative systemic treatment	2	Yes (75)
When a gynecological sarcoma is diagnosed, the case must be referred to an interdisciplinary team for agreement on what type of treatment would be best for the individual patient	2	Yes (75)

**Table 7:** Recommendations for disease follow-up.

Recommendation	Phase	Type of consensus (% agreement)
Patients with excised low-grade STS who had negative resection margins should have		
(i) Local MRI every 6 months during the first 2 years, as well as a physical exam every 3 or 6 months if considered appropriate	2	Yes (mode: 50)
(ii) Physical exam every 6 months and local MRI annually from 3rd to 5th year	2	Yes (mode: 68.3)
(iii) Physical exam and local MRI annually from 6th to 10th year	2	Yes (mode: 68.3)
(iv) No follow-up is necessary after 10th year	1	Yes (mode: 65)
Patients with low-grade resected STS with focally positive margins that cannot be widened should have the following:		
(i) Physical exam and local MRI every 3-4 months during the first 2 years	1	Yes (mode: 70)
(ii) Physical exam and local MRI every 6 months from 3rd to 5th year	1	Yes (mode: 65)
(iii) Physical exam and local MRI annually from 6th to 10th year	1	Yes (mode: 70)
(iv) No imaging tests are necessary after 10th year, and no further follow-up can be considered vs. annual physical exam	2	—
Patients with high-risk (>5 cm, deep-seated, and high-grade) resected STS with focally positive margins that cannot be widened and who have only received complementary radiation therapy should have the following:		
(i) Physical exam and local MRI every 3-4 months during the first 2 years	1	Yes (mode: 80)
(ii) Physical exam and local MRI every 6 months from 3rd to 5th year	1	Yes (mode: 80)
(iii) Physical exam and local MRI annually from 6th to 10th year	1	Yes (mode: 85)
(iv) No imaging tests are necessary after 10th year, and no further follow-up can be considered vs. annual physical exam	2	—
Patients with (<5 cm, deep-seated, and high-grade) resected STS with focally positive margins that cannot be widened and who have only received complementary radiation therapy should have the following:		
(i) Physical exam and local MRI every 3-4 months during the first 2 years	1	Yes (mode: 80)
(ii) Physical exam and local MRI every 6 months from 3rd to 5th year	1	Yes (mode: 75)
(iii) Physical exam and local MRI annually from 6th to 10th year	1	Yes (mode: 75)
(iv) No imaging tests are necessary after 10th year, and no further follow-up can be considered	2	Yes (mode: 56)
Patients with localized intermediate-high-grade STS should have a chest CT scan every 3-4 months during the first 2 years, every 6 months from 3rd to 5th year, and thereafter annually until 10th year	1	Yes (mode: 70)
After resection of the pulmonary metastases, patients who have had metastatic disease should have a lung CT scan every 3 months during the first 2 years and subsequently every 6 months	1	Yes (80)
Factors to be taken into account to consider that a patient has progressed on a treatment and needs to change to a different one are as follows(in decreasing order of importance):		
(i) Clinical progression	2	Yes (mean: 3.3; CV: 23.9%)
(ii) Progression based on RECIST criteria	2	Yes (mean: 3.1; CV: 25.2%)
PET-CT scans are considered especially useful prior to the resection of pulmonary metastases from STS	2	Yes (75)

## Appendix

- Diego Soto de Prado
- Ana Sebio
- Josep Piera
- Pablo Luna
- Jerónimo Martínez
- Anna Estival
- J. Martínez Trufero
- Alberto Moreno
- Nadia Hindi
- Juana Cano
- María Ángeles Vaz
- Isabel Sevilla
- Carlos Álvarez
- Andrés Redondo
- Luis de Sande
- César Serrano
- Roberto Díaz
- J. Martín Algarra
- Andrés Meana
- Ana de Juan

## References

[1] Loong H. H., Wong K. H., Tse T. (2018). Controversies and consensus of neoadjuvant chemotherapy in soft-tissue sarcomas. *ESMO Open*.

[2] Casali P. G., Abecassis N., Bauer S. (2018). ESMO Guidelines Committee and EURACAN. Soft tissue and visceral sarcomas: ESMO-EURACAN Clinical Practice Guidelines for diagnosis, treatment and follow-up. *Annals of Oncology*.

[3] Noone A.-M., Cronin K. A., Altekruse S. F. (2017). Cancer incidence and survival trends by subtype using data from the surveillance epidemiology and end results program, 1992-2013. *Cancer Epidemiology Biomarkers and Prevention*.

[4] Maretty-Nielsen K., Aggerholm-Pedersen N., Keller J. (2014). Relative mortality in soft tissue sarcoma patients: a Danish population-based cohort study. *BMC Cancer*.

[5] European Society for Medical Oncology (ESMO)/European Sarcoma Network Working Group (2014). Soft tissue and visceral sarcomas: ESMO clinical practice guidelines for diagnosis, treatment and follow-up. *Annals of Oncology*.

[6] Hudgens S., Forsythe A., Kontoudis I. (2017). Evaluation of quality of life at progression in patients with soft tissue sarcoma. *Sarcoma*.

[7] Grupo Español de Investigación en Sarcomas (2006). Guías Clínicas en Sarcoma de Partes Blandas. *Oncología*.

[8] García Del Muro X., Martín J., Maurel J I. (2011). Guía de práctica clínica en los sarcomas de partes blandas. *Medicina Clínica*.

[9] von Mehren M., Randall R. L., Benjamin R. S. (2018). Soft tissue sarcoma, version 2.2018, NCCN clinical practice guidelines in oncology. *Journal of the National Comprehensive Cancer Network*.

[10] Dalkey N. C. (1969). *The Delphi Method: An Experimental Study of Group Opinion*.

[11] Gronchi A., Stacchiotti S., Verderio P. (2016). Short, full-dose adjuvant chemotherapy (CT) in high-risk adult soft tissue sarcomas (STS): long-term follow-up of a randomized clinical trial from the Italian Sarcoma Group and the Spanish Sarcoma Group. *Annals of Oncology*.

[12] Trojani M., Contesso G., Coindre J. M. (1984). Soft-tissue sarcomas of adults; study of pathological prognostic variables and definition of a histopathological grading system. *International Journal of Cancer*.

[13] Tanaka K., Mizusawa J., Fukuda H. (2015). Perioperative chemotherapy with ifosfamide and doxorubicin for high-grade soft tissue sarcomas in the extremities (JCOG0304). *Japanese Journal of Clinical Oncology*.

[14] Pasquali S., Gronchi A. (2017). Neoadjuvant chemotherapy in soft tissue sarcomas: latest evidence and clinical implications. *Therapeutic Advances in Medical Oncology*.

[15] Gronchi A., Ferrari S., Quagliuolo V. (2017). Histotype-tailored neoadjuvant chemotherapy versus standard chemotherapy in patients with high-risk soft-tissue sarcomas (ISG-STS 1001): an international, open-label, randomised, controlled, phase 3, multicentre trial. *The Lancet Oncology*.

[16] Kandel R., Coakley N., Werier J. (2013). Surgical margins and handling of soft-tissue sarcoma in extremities: a clinical practice guideline,. *Current Oncology*.

[17] Kumar V., Misra S., Chaturvedi A. (2012). Retroperitoneal sarcomas—a challenging problem. *Indian Journal of Surgical Oncology*.

[18] Hager S., Makowiec F., Henne K. (2017). Significant benefits in survival by the use of surgery combined with radiotherapy for retroperitoneal soft tissue sarcoma. *Radiation Oncology*.

[19] Kang S., Kim H.-S., Kim W., Kim J. H., Kang S. H., Han I. (2015). Comorbidity is independently associated with poor outcome in extremity soft tissue sarcoma. *Clinics in Orthopedic Surgery*.

[20] Lehnhardt M., Muehlberger T., Kuhnen C. (2005). Feasibility of chemosensitivity testing in soft tissue sarcomas. *World Journal of Surgical Oncology*.

[21] Puri A., Gulia A. (2011). Management of extremity soft tissue sarcomas. *Indian Journal of Orthopaedics*.

[22] Hoven-Gondrie M. L., Bastiaannet E., Ho V. K. Y. (2016). Worse survival in elderly patients with extremity soft-tissue sarcoma. *Annals of Surgical Oncology*.

[23] Linch M., Miah A. B., Thway K., Judson I. R., Benson C. (2014). Systemic treatment of soft-tissue sarcoma-gold standard and novel therapies. *Nature Reviews Clinical Oncology*.

[24] Judson I., Verweij J., Gelderblom H. (2014). Doxorubicin alone versus intensified doxorubicin plus ifosfamide for first-line treatment of advanced or metastatic soft-tissue sarcoma: a randomised controlled phase 3 trial. *The Lancet Oncology*.

[25] Tap W. D., Jones R. L., Van Tine B. A. (2016). Olaratumab and doxorubicin versus doxorubicin alone for treatment of soft-tissue sarcoma: an open-label phase 1b and randomised phase 2 trial. *The Lancet*.

[26] Stacchiotti S., Negri T., Zaffaroni N. (2011). Sunitinib in advanced alveolar soft part sarcoma: evidence of a direct antitumor effect. *Annals of Oncology*.

[27] Judson I., Morden J. P., kilburn l. (2019). Cediranib in patients with alveolar soft-part sarcoma (CASPS): a double-blind, placebo-controlled, randomised, phase 2 trial. *The Lancet Oncology*.

[28] Desar I. M., Constantinidou A., Kaal S. E. (2016). Advanced soft-tissue sarcoma and treatment options: critical appraisal of trabectedin. *Cancer Management and Research*.

[29] G. K., Hu J. S., Tseng W. W. (2017). Treatment of advanced, metastatic soft tissue sarcoma: latest evidence and clinical considerations. *Therapeutic Advances in Medical Oncology*.

[30] Sanfilippo R., Dileo P., Blay J. Y. (2015). Trabectedin in advanced synovial sarcomas a multicenter retrospective study from four European institutions and the Italian Rare Cancer Network. *Anti-Cancer Drugs*.

[31] Larsen A. K., Galmarini C. M., D’Incalci M. (2016). Unique features of trabectedin mechanism of action. *Cancer Chemotherapy and Pharmacology*.

[32] Le Cesne A., Ray-Coquard I., Duffaud F. (2015). Trabectedin in patients with advanced soft tissue sarcoma: a retrospective national analysis of the French Sarcoma Group. *European Journal of Cancer*.

[33] Sharma S., Takyar S., Manson S. C. (2013). Efficacy and safety of pharmacological interventions in second or later-line treatment of patients with advanced soft tissue sarcoma a systematic review. *BMC Cancer*.

[34] Tascilar M., Loos W. J., Seynaeve C., Verweij J., Sleijfer S. (2007). The pharmacologic basis of ifosfamide use in adult patients with advanced soft tissue sarcomas. *The Oncologist*.

[35] Sleijfer S., Ouali M., van Glabbeke M. (2010). Prognostic and predictive factors for outcome to first-line ifosfamide-containing chemotherapy for adult patients with advanced soft tissue sarcomas. *European Journal of Cancer*.

[36] Wilky B. A., Meyer C. F., Trent J. C. (2013). Pazopanib in sarcomas. *Current Opinion in Oncology*.

[37] Powles T., Bracarda S., Chen M. (2015). Characterisation of liver chemistry abnormalities associated with pazopanib monotherapy: a systematic review and meta-analysis of clinical trials in advanced cancer patients. *European Journal of Cancer*.

[38] van der Graaf W. T., Blay J.-Y., Chawla S. P. (2012). Pazopanib for metastatic soft-tissue sarcoma (PALETTE): a randomised, double-blind, placebo-controlled phase 3 trial. *The Lancet*.

[39] Schöffski P., Chawla S., Maki R. G. (2016). Eribulin versus dacarbazine in previously treated patients with advanced liposarcoma or leiomyosarcoma: a randomised, open-label, multicentre, phase 3 trial. *The Lancet*.

[40] Thomas C., Movva S. (2016). Eribulin in the management of inoperable soft-tissue sarcoma: patient selection and survival. *OncoTargets and Therapy*.

[41] Losa R., Fra J., López-Pousa A. (2007). Phase II study with the combination of gemcitabine and DTIC in patients with advanced soft tissue sarcomas. *Cancer Chemotherapy and Pharmacology*.

[42] García-del-Muro X., López-Pousa A., Maurel J. (2011). Randomized phase II study comparing gemcitabine plus dacarbazine versus dacarbazine alone in patients with previously treated soft tissue sarcoma: a Spanish Group for Research on Sarcomas study. *Journal of Clinical Oncology*.

[43] Ducoulombier A., Cousin S., Kotecki N., Penel N. (2016). Gemcitabine-based chemotherapy in sarcomas: a systematic review of published trials. *Critical Reviews in Oncology/Hematology*.

[44] Maki R. G., Wathen J. K., Patel S. R. (2007). Randomized phase II study of gemcitabine and docetaxel compared with gemcitabine alone in patients with metastatic soft tissue sarcomas: results of sarcoma alliance for Research through collaboration study 002. *Journal of Clinical Oncology*.

[45] Grimer R., Judson I., Peake D., Seddon B. (2010). Guidelines for the management of soft tissue sarcomas. *Sarcoma*.

[46] Ferguson P. C., Deheshi B. M., Chung P. (2011). Soft tissue sarcoma presenting with metastatic disease. *Cancer*.

[47] Jiang L., Jiang S., Lin Y. (2015). Significance of local treatment in patients with metastatic soft tissue sarcoma. *American Journal of Cancer Research*.

[48] Stragliotto C. L., Karlsson K., Lax I. (2012). A retrospective study of SBRT of metastases in patients with primary sarcoma. *Medical Oncology*.

[49] Lindsay A. D., Haupt E. E., Chan C. M. (2018). Treatment of sarcoma lung metastases with stereotactic body radiotherapy. *Sarcoma*.

[50] Treasure T., Fiorentino F., Scarci M., Møller H., Utley M. (2012). Pulmonary metastasectomy for sarcoma: a systematic review of reported outcomes in the context of Thames Cancer Registry data. *BMJ Open*.

[51] van Houdt W. J., Zaidi S., Messiou C., Thway K., Strauss D. C., Jones R. L. (2017). Treatment of retroperitoneal sarcoma. *Current Opinion In Oncology*.

[52] Bonvalot S., Gronchi A., Pechoux C. L. (2019). STRASS (EORTC 62092): a phase III randomized study of preoperative radiotherapy plus surgery versus surgery alone for patients with retroperitoneal sarcoma. *Journal of Clinical Oncology*.

[53] Datta J., Ecker B. L., Neuwirth M. G. (2017). Contemporary reappraisal of the efficacy of adjuvant chemotherapy in resected retroperitoneal sarcoma: evidence from a nationwide clinical oncology database and review of the literature. *Surgical Oncology*.

[54] Thanopoulou E., Aleksic A., Thway K. (2015). Hormonal treatments in metastatic endometrial stromal sarcomas: the 10-year experience of the sarcoma unit of Royal Marsden Hospital. *Clinical Sarcoma Research*.

[55] Cheng X., Yang G., Schmeler K. M. (2011). Recurrence patterns and prognosis of endometrial stromal sarcoma and the potential of tyrosine kinase-inhibiting therapy. *Gynecologic Oncology*.

[56] Thanopoulou E., Judson I. (2012). Hormonal therapy in gynecological sarcomas. *Expert Review of Anticancer Therapy*.

[57] Yamaguchi M., Erdenebaatar C., Saito F. (2015). Long-term outcome of aromatase inhibitor therapy with letrozole in patients with advanced low-grade endometrial stromal sarcoma. *International Journal of Gynecologic Cancer*.

[58] El-Khalfaoui K., du Bois A., Heitz F., Kurzeder C., Sehouli J., Harter P. (2014). Current and future options in the management and treatment of uterine sarcoma. *Therapeutic Advances in Medical Oncology*.

[59] Reed N. S. (2008). The management of uterine sarcomas. *Clinical Oncology*.

[60] Kim W. Y., Lee J.-W., Choi C. H. (2008). Low-grade endometrial stromal sarcoma: a single center’s experience with 22 cases. *International Journal of Gynecologic Cancer*.

[61] Feng W., Hua K., Malpica A., Zhou X., Baak J. P. A. (2013). Stages I to II WHO 2003-defined low-grade endometrial stromal sarcoma: how much primary therapy is needed and how little is enough?. *International Journal of Gynecologic Cancer*.

[62] (2019). National comprehensive cancer network clinical practice guidelines: soft tissue sarcoma. https://www.nccn.org/store/login/login.aspx?ReturnURL=https://www.nccn.org/professionals/physician_gls/PDF/sarcoma.pdf.

[63] Dinh T. A., Oliva E. A., Fuller A. F., Lee H., Goodman A. (2004). The treatment of uterine leiomyosarcoma. Results from a 10-year experience (1990–1999) at the Massachusetts General Hospital. *Gynecologic Oncology*.

[64] Seagle B.-L. L., Sobecki-Rausch J., Strohl A. E., Shilpi A., Grace A., Shahabi S. (2017). Prognosis and treatment of uterine leiomyosarcoma: a National Cancer Database study. *Gynecologic Oncology*.

[65] Park J.-Y., Kim D.-Y., Suh D.-S. (2008). Prognostic factors and treatment outcomes of patients with uterine sarcoma: analysis of 127 patients at a single institution, 1989–007. *Journal of Cancer Research and Clinical Oncology*.

[66] Amant F., Lorusso D., Mustea A. (2015). Management strategies in advanced uterine leiomyosarcoma: focus on trabectedin. *Sarcoma*.

[67] Kapp D. S., Shin J. Y., Chan J. K. (2008). Prognostic factors and survival in 1396 patients with uterine leiomyosarcomas. *Cancer*.

[68] Sawamura C., Matsumoto S., Shimoji T., Tanizawa T., Ae K. (2012). What are risk factors for local recurrence of deep high-grade soft-tissue sarcomas?. *Clinical Orthopaedics and Related Research*.

[69] Sawamura C., Matsumoto S., Shimoji T., Okawa A., Ae K. (2014). How long should we follow patients with soft tissue sarcomas?. *Clinical Orthopaedics and Related Research*.

[70] Hoshi M., Oebisu N., Takada J., Ieguchi M., Wakasa K., Nakamura H. (2014). Role of FDG-PET/CT for monitoring soft tissue tumors. *Oncology Letters*.

[71] Chen L., Wu X., Ma X., Guo L., Zhu C., Li Q. (2017). Prognostic value of 18F-FDG PET-CT-based functional parameters in patients with soft tissue sarcoma. *Medicine*.

